# Pediatric endoscopic subcutaneous mastectomy (pesma) with liposuction in adolescents with gynecomastia

**DOI:** 10.1007/s00464-022-09550-x

**Published:** 2022-09-01

**Authors:** François Varlet, Ciro Esposito, Aurelien Scalabre, Benedetta Lepore, Sophie Vermersch, Maria Escolino

**Affiliations:** 1 Division of Pediatric Surgery, Centre Hospitalier Universitaire (CHU) de Saint-Etienne, Saint-Etienne, France; 2 Division of Pediatric Surgery, Federico II University Hospital, Via Pansini 5, 80131 Naples, Italy

**Keywords:** Gynecomastia, Endoscopic, Liposuction, Technique, Adolescents, MIS

## Abstract

**Background:**

Surgical techniques for treatment of gynecomastia are increasingly less invasive. We described technical standardization of pediatric endoscopic subcutaneous mastectomy (PESMA) with liposuction.

**Methods:**

All adolescents with primary gynecomastia, operated using PESMA with liposuction over the period June 2014–July 2021, were included. The video recording of procedures was analyzed to standardize the operative technique. After patient installation, 3 trocars were placed on the mid-axillary line. The technique included 5 steps: (1) subcutaneous injection of lipolysis solution and liposuction; (2) creation of working space using an inflated balloon; (3) gland dissection using 5-mm sealing device; (4) specimen extraction through the largest trocar orifice; and (5) placement of suction drainage tube.

**Results:**

Twenty-four male adolescents, operated for Simon’s grade 2B and 3 gynecomastia using PESMA with liposuction over the study period, were included. Mean patient age was 16 years (range 15–18). Gynecomastia was bilateral in 19/24 (79.2%) and unilateral in 5/24 (20.8%). One (4.1%) conversion to open was reported. The mean operative time was 87 min (range 98–160) for unilateral and 160 min (range 140–250) for bilateral procedure. The mean length of stay was 2.2 days (range 1–4). Patients wore a thoracic belt for 15 up to 30 days postoperatively. Post-operative complications occurred in 5/24 (20.8%): 2- or 3 mm second-degree burns in 4 (16.7%) and subcutaneous seroma in 1 (4.1%). All complications were Clavien 2 grade and did not require further treatment. Aesthetic outcomes were very good in 21/24 (87.5%). Three (12.5%) boys had persistent minimal breast asymmetry but did never perceive it negatively.

**Conclusion:**

PESMA combined with liposuction was feasible and safe for surgical treatment of gynecomastia in this selected cohort of patients. Although challenging, this procedure provided good aesthetic results, with no scars on the anterior thoracic wall. Standardization of the operative technique was a key point for successful outcome.

**Supplementary Information:**

The online version contains supplementary material available at 10.1007/s00464-022-09550-x.

Gynecomastia is defined as an abnormal enlargement of one or two breasts in male, caused by benign proliferation of the gland ducts and stromal components including fat [1]. It represents the most common breast swelling in males [2], with a prevalence rate higher than 60% in adolescents and even greater in obese patients [3, 4]. The main etiology of gynecomastia occurring during pubertal development is referred to transient altered estrogen-to-androgen ratio and this condition is named idiopathic or primary gynecomastia [5]. Pubertal gynecomastia is self-limited in up to 90% of adolescents and regresses spontaneously in a variable period from 6 months to 3 years [6]. Hence, in most of such cases, observation and reassurance without specific treatments are usually enough [1]. On the other hand, several conditions such as endocrine tumors and/or dysfunctions, liver failure, chronic kidney diseases, and drugs may induce secondary gynecomastia [7]. Treating the underlying disease or discontinuing medications might resolve gynecomastia in such cases.

Nevertheless, the persistence of gynecomastia may cause significant psychological consequences in the affected adolescents, which include depression, anxiety, deterioration of self-esteem, and reduced quality of life [8, 9]. Therefore, in boys with persistent gynecomastia causing significant psychosocial distress, surgical treatment could be considered. The most adopted classification of gynecomastia is Simon’s grading scale [10]. Patients with a gynecomastia corresponding to a Simon’s grade 2B and 3 should be addressed to surgical correction. Analyzing the international literature, subcutaneous mastectomy through a trans- or peri-areolar access has been initially the most adopted technique to correct this condition, although it may cause poor scarring and asymmetry [11–13]. Thus, minimally invasive techniques have been progressively introduced with the double aim of removing the fatty tissue using liposuction and performing a video-assisted excision of the gland [14]. Endoscopic subcutaneous mastectomy (ESCM) was reported in adult series and this technique was considered safe and effective with good aesthetic results [15–17]. The first pediatric experience using the endoscopic approach was described in 2019 in 12 patients [18].

Over the last year, we standardized the operative technique modifying some technical details to reduce the length of surgery and improve the aesthetic results. This paper aimed to describe the standardization of surgical technique of pediatric endoscopic subcutaneous mastectomy (PESMA) with liposuction in adolescents with gynecomastia.

## Materials and methods

Open surgery with liposuction and PESMA with liposuction were fully presented and explained to consecutive patients with primary gynecomastia in two pediatric surgery units. All adolescents, who chose to be operated on using PESMA and liposuction in the period June 2014–July 2021, were included in the study.

Exclusion criteria were patients already operated for gynecomastia or with a secondary gynecomastia requiring medical or symptomatic treatment.

Data were retrospectively collected by analysis of patients’ records, follow-up evaluations, and patient interviews. Patients’ records were reviewed regarding degree of gynecomastia according to Simon’s classification [10], operative technique details, relapses, and complications (wound healing disorders, numbness).

During the follow-up, the aesthetic outcome assessment was done by two independent surgeons, who were not involved in the surgical procedures and did not have any information about the patients. In detail, they documented the condition of the scars, any breast asymmetry, and/or nipple retraction. Additionally, patients were asked to evaluate subjectively the overall aesthetic outcome on a Likert-type scale from 1 to 5 (1 = very bad; 2 = bad; 3 = acceptable; 4 = good; 5 = very good).

**Fig. 1:**
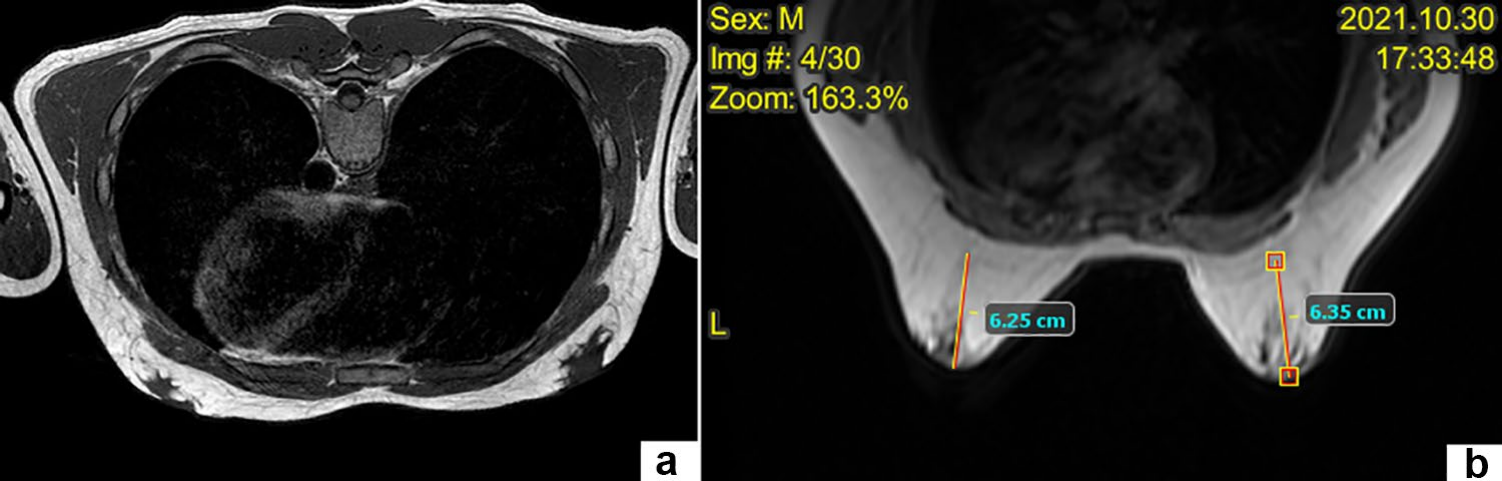
MRI was helpful to differentiate gynecomastia (**a**) from adipomastia (**b**)

Pre-operatively, all patients received an endocrinological consultation to exclude diagnosis of hypogonadism or other secondary causes for gynecomastia. Also, a careful physical examination including the genitourinary system was conducted to identify signs of hypogonadism or testicular tumors. Pre-operative work-up included standard ultrasonography of the breasts to evaluate the local anatomy. Over the last years, we also performed a magnetic resonance imaging (MRI) without contrast with the aim to evaluate, in the transverse and sagittal plans and in T2 sequences, the amount of fatty tissue and the aspect, size, and morphology of the gland. MRI was also useful to differentiate gynecomastia from adipomastia or adipo-gynecomastia and plan the surgical strategy (Fig. 1).

The study received the appropriate institute review board (IRB) approval. All patients gave their written consent to publish photographs and data.

### Indications for surgery

Surgical indications for adolescent gynecomastia in both institutions were patients with persistent and/or progressive gynecomastia lasting more than 3 years, with accompanying psychosocial distress or symptoms such as persistent pain.

### Operative technique

**Fig. 2:**
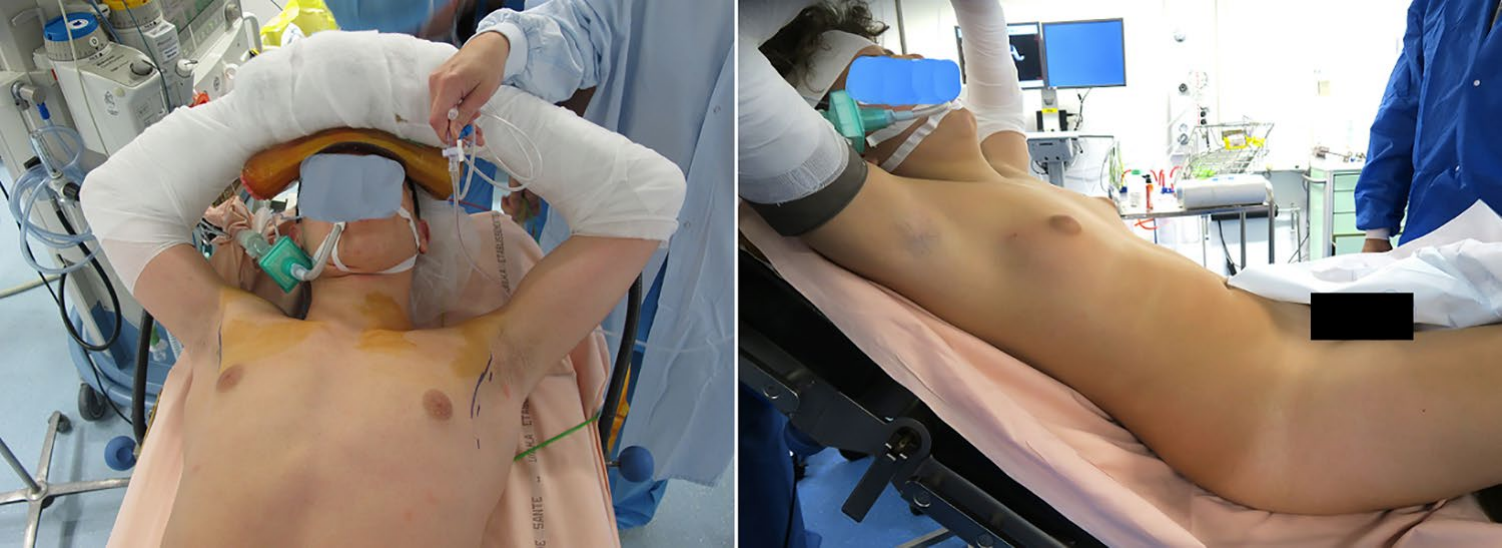
Patient installation

**Fig. 3:**
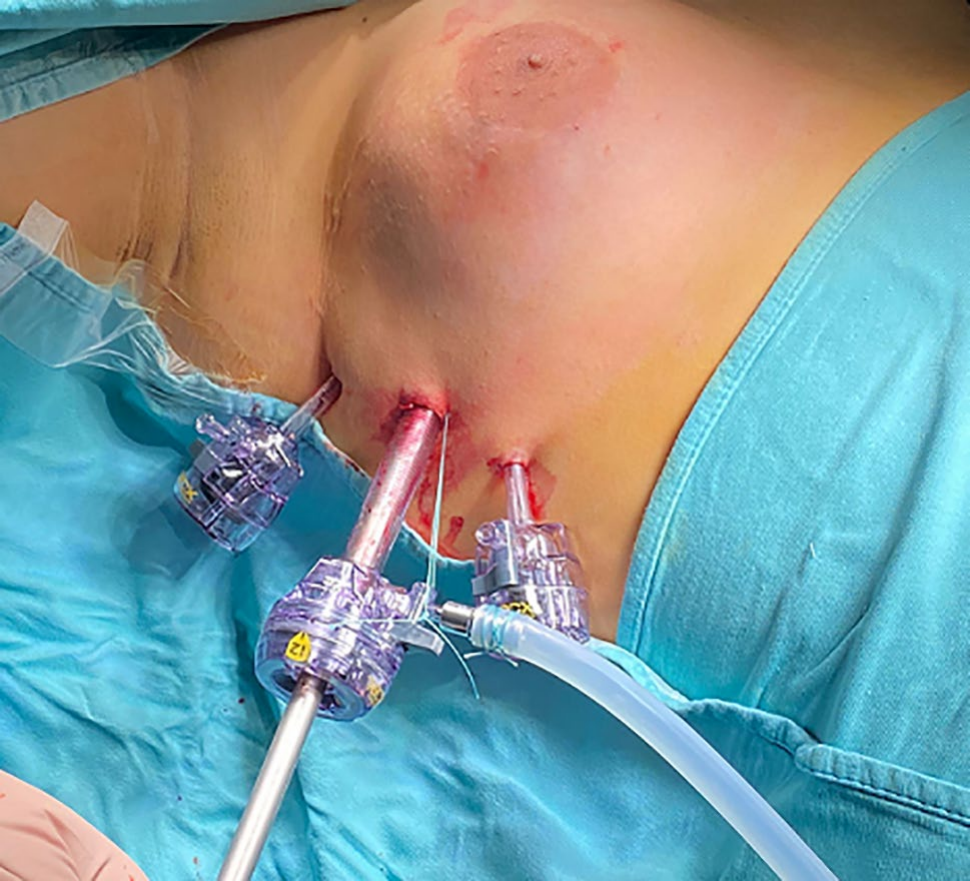
Port positioning

**Fig. 4:**
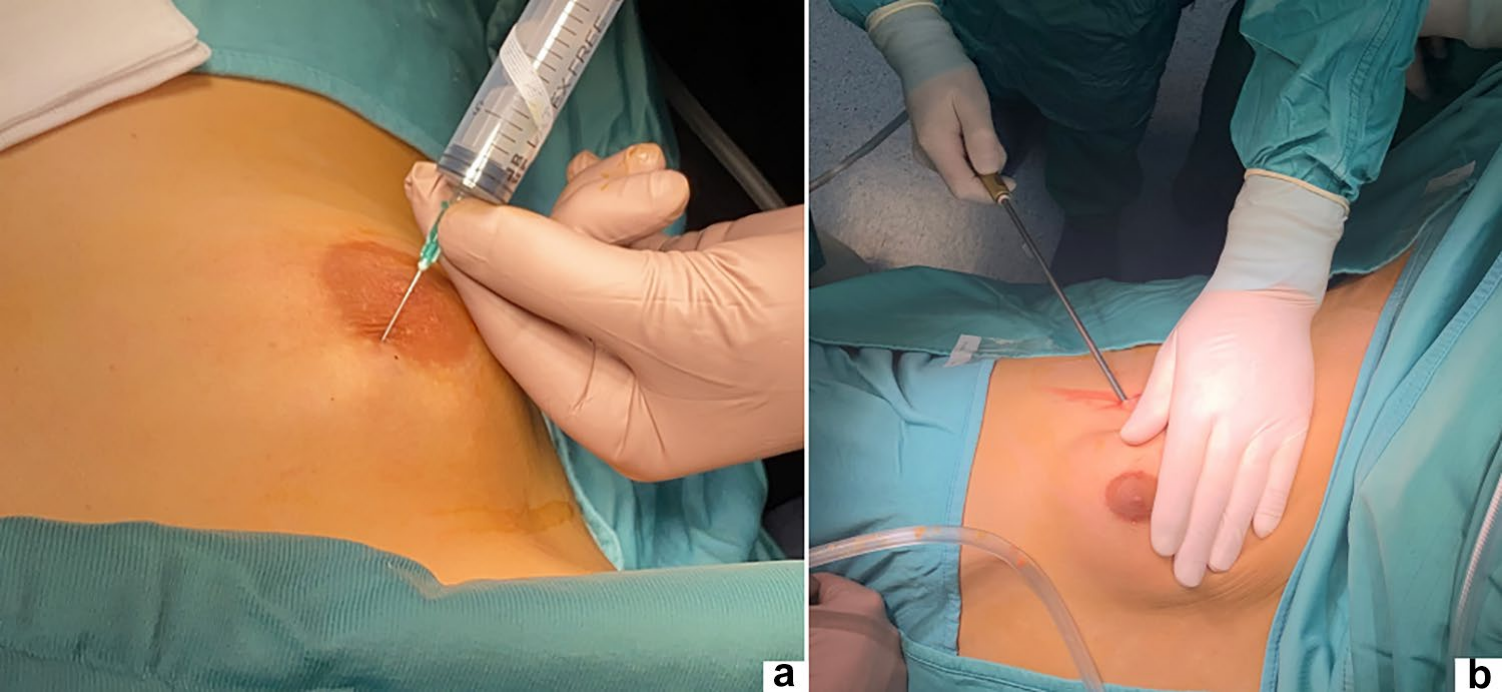
After subcutaneous injection of lipolysis solution (**a**), liposuction is performed using a 5 mm device (**b**)

All patients were operated under general anesthesia using endoscopic approach (PESMA). Patient installation was very important; the patient was placed in semi-seated position with the ipsilateral arm, or both the arms abducted at 145 degrees and fixed on headframe (Fig. 2). Three trocars were placed on the mid-axillary line: one 12- or 10-mm port for 30 degree optic, centrally located on the same axis of the nipple, and two lateral 5-mm working ports, aligned with the optic port and at 2–3 cm away from it (Fig. 3). In the last year, we have standardized the technique in 5 steps:

Step 1: A lipolysis solution, containing 200 mL of sterile water, 200 mL of saline, 20 mL of 2% lidocaine, and 1 mL of 0.1% adrenaline, was injected subcutaneously around the gland, not too close to the skin and nipple area. The amount of injected solution was about 100–200 mL but varied according to the patient size and gynecomastia size. After that, the working space was first created using a 5-mm liposuction aspiration device (Fig. 4). The endpoint of the liposuction was decided by the pinch thickness of breast tissue when the fibrous gland could be clearly seen and felt. This phase lasted about 5–15 min and aimed to remove the fatty tissue partially responsible of the breast enlargement.

**Fig. 5:**
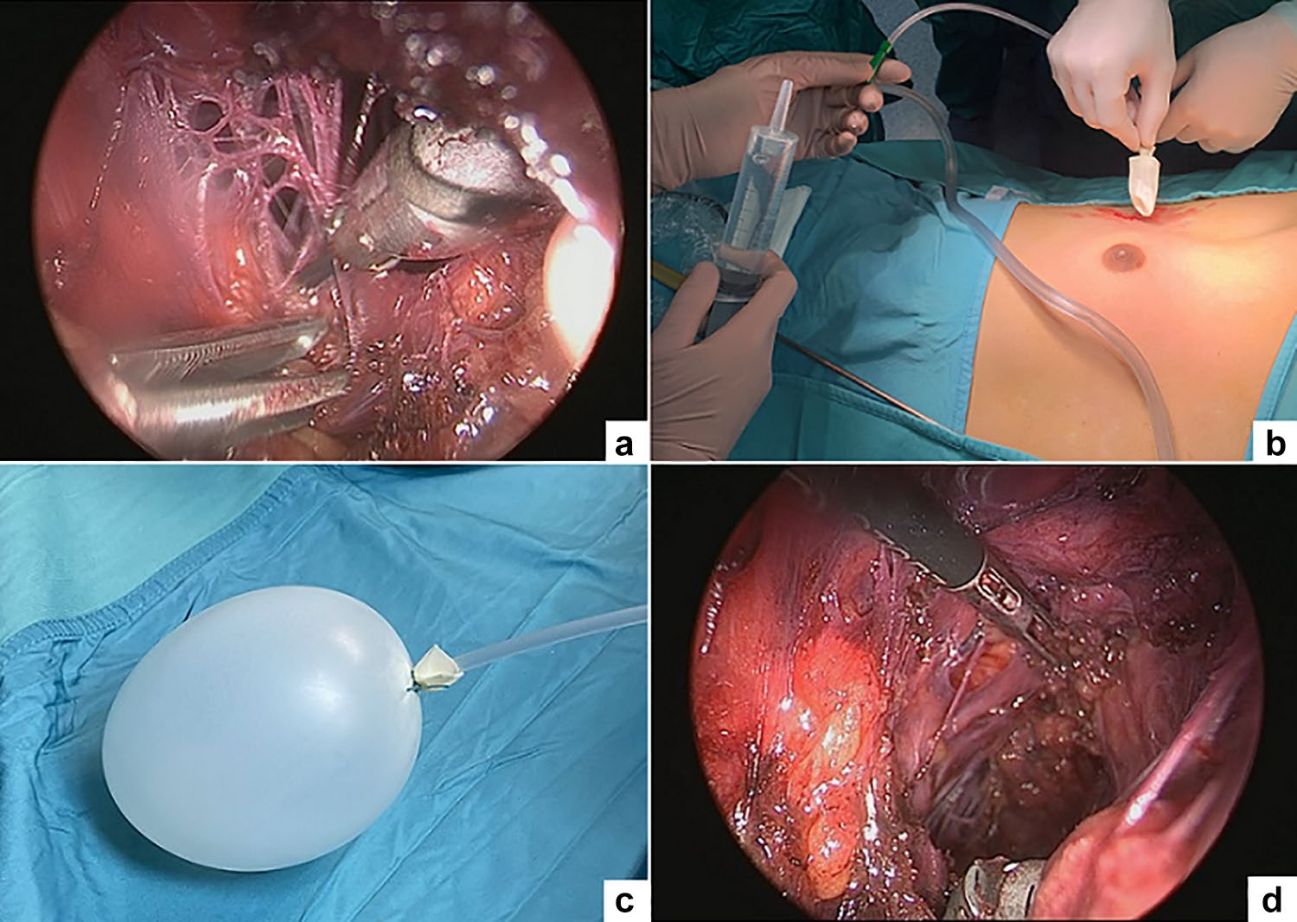
After liposuction, the working space is not free with a lot of bands (Cooper ligament) (**a**), the home-made glove balloon device is inserted into the cavity (**b**) and inflated with saline (**c**) to favor Cooper ligament collapse (**d**)

Step 2: After liposuction, the working space between the gland and pectoralis muscle was not yet free, with a lot of bands (Cooper ligament). The creation of the working space was completed by introducing into the cavity a balloon inflated with saline (Fig. 5). This balloon was created by ligating a finger glove on the tip of a 16F Nelaton catheter, similarly to that Gaur previously described for retroperitoneoscopy [19].

Step 3: After insufflation of CO_2_ with 8 mmHg pressure, subcutaneous dissection of the gland was performed up to the lateral margin of the pectoralis major muscle, using 5-mm Harmonic Scalpel (Ethicon, Somerville, NJ). During this phase, the operating surgeon kept nondominant hand on the nipple to control the thickness of the flap and feel the tip of the sealing device through the transilluminated skin, to prevent nipple-areolar complex (NAC) or skin injury. When the dissection was completed, the gland resection was performed leaving about 5–10 mm of gland thickness behind the NAC to avoid nipple retraction.

**Fig. 6:**
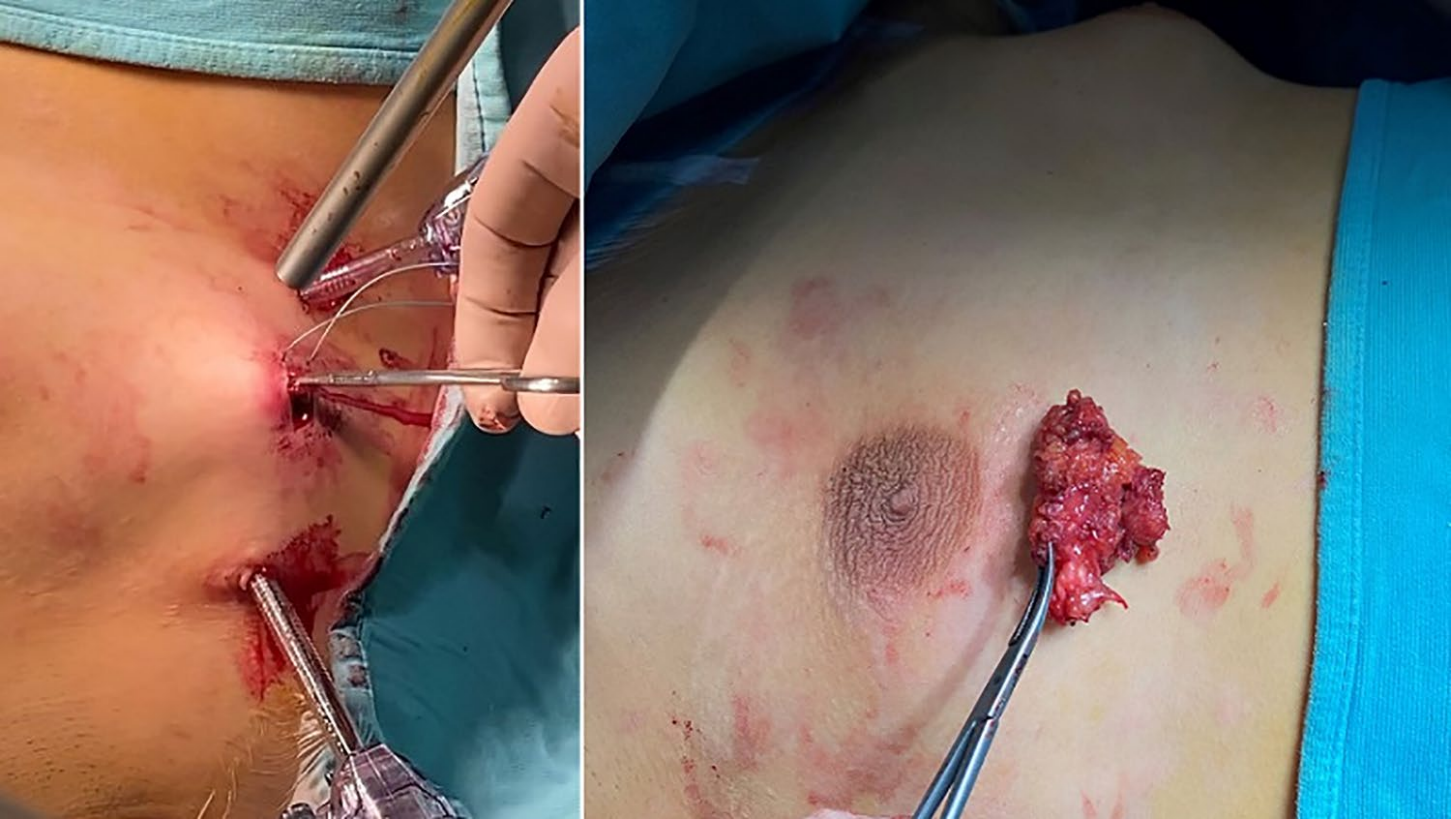
Specimen extraction through the largest trocar orifice

Step 4: The gland was removed through the largest trocar orifice (10 mm or 12 mm), as a whole or after cutting it into strip-like pieces before extraction (Fig. 6).

Step 5: After checking the hemostasis under vision, a suction drainage tube was placed in each cavity through one 5-mm trocar orifice. The trocars’ incisions were closed by intermittent suture for subcutaneous tissue and intradermal continuous suture with absorbable sutures. The resected tissues in all operated patients were sent for histopathology.

All steps of PESMA technique are summarized in Video 1.

### Post-operative course

**Fig. 7:**
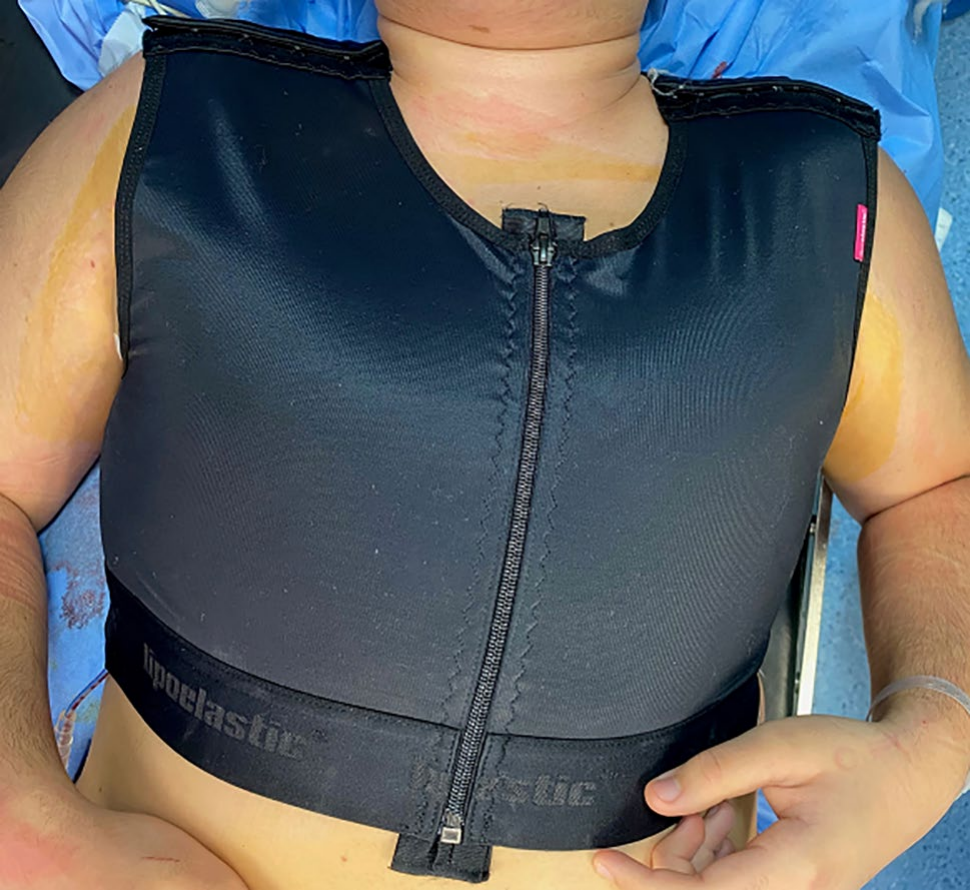
The patients wore a compressive thoracic belt for 15 up to 30 days postoperatively

The suction drainage tube was removed when the output volume was less than 40 mL/day. The patients were instructed to wear a thoracic belt smoothly compressing the operative areas for 15 up to 30 days postoperatively (Fig. 7).

The patients were reviewed at POD 7, 30, 90, 180 and thereafter annually for 2 years postoperatively, with clinical examination, pictures, and assessment of patients’ satisfaction. The result was considered as good when there was no residual gland, good symmetry, and no nipple retraction or lateral displacement, and bad in case of residual gland, breast asymmetry, and nipple displacement or retraction.

### Statistical analysis

Statistical analysis was carried out using the Statistical Package for Social Sciences (SPSS Inc., Chicago, Illinois, USA), version 13.0. Continuous variables were summarized and presented as median and interquartile range. The categorical variables were presented as absolute numbers and percentages. The associations between quantitative variables were measured using the parametric Student’s *t* test. *P* value < 0.05 was considered statistically significant.

## Results

### Patient characteristics

Twenty-four adolescents with primary gynecomastia were operated using PESMA with liposuction in the study period and included. The mean patient age was 16 years (range 15–18), and the mean patient weight was 72 kg (range 59–95). The mean body mass index (BMI) was 21.3 (range 17–26). Eight patients (33.3%) were either overweight or obese. Weight control was either refused or failed in most of patients (*n* = 6, 75%). Even when they successfully reduced their weight (*n* = 2, 25%), the change in the size of the breasts was not satisfactory and they requested surgical correction. At the time of pre-operative evaluation, no one complained of breast pain or abnormal nipple discharge. Pathology was bilateral in 19 (79.2%) and unilateral in 5 (20.8%). The appearance of the breast was categorized as Simon’s grade 2b in 16 (66.7%) and Simon’s grade 3 in 8 (33.3%).

Patient characteristics are reported in Table 1.

**Table 1:** Patient characteristics

Series number, *n*	24
Mean age, years (range)	16 (15–18)
Mean weight, kg (range)	72 (59–95)
Mean BMI, kg/m^2^ (range)	21.3 (17–26)
Bilateral pathology, *n* (%)	19 (79.2%)
Unilateral pathology, *n* (%)	5 (20.8%)
Grade according to Simon’s classification	
1, *n* (%)	0
2a, *n* (%)	0
2b, *n* (%)	16 (66.7%)
3, *n* (%)	8 (33.3%)

*BMI* body mass index

### Operative results, complications, aesthetic outcomes

The mean operative time was 87 min (range 98–160) for unilateral and 160 min (range 140–250) for bilateral procedure. The length of surgery improved with learning curve and use of sealing devices to perform dissection. In the last seven patients, in whom the inflated balloon was adopted to create the working space, the mean operative time for bilateral procedure diminished to 135 min (*p* = 0.001). A conversion to open was reported in one bilateral case (4.1%) at beginning of experience, due to excessive length of surgery and bad endoscopic vision with 5 mm laparoscope.

The mean weight of resected fibrous glandular tissue of breasts was 48.3 g (range 10–97), and the mean aspirated volume of chest fat was 240 mL (range 70–400). The mean volume of intra-operative blood loss was 15.5 mL (range 5–60). The mean drainage time post-surgery was 1.8 days (range 1–3), and the mean drainage volume post-surgery was 68.5 mL (range 15–210), which mostly appeared on the first day post-surgery.

The mean length of stay was 2.2 days (range 1–4), and the follow-up period ranged from 12 to 24 months. Histopathology confirmed diagnosis of glandular hyperplasia in all patients, and no malignant pathology was discovered.

Post-operative complications occurred in 5/24 (20.8%): 2- or 3-mm second-degree burns in 4 (16.7%), located on the nipple (*n* = 2) or just above the nipple (*n* = 2), with a 2-mm residual scar above the nipple. A subcutaneous seroma occurred in 1 (4.1%) and disappeared after 3 weeks of prolonged compression by thoracic belt. All complications were Clavien 2 grade and did not require further treatment.

**Fig. 8:**
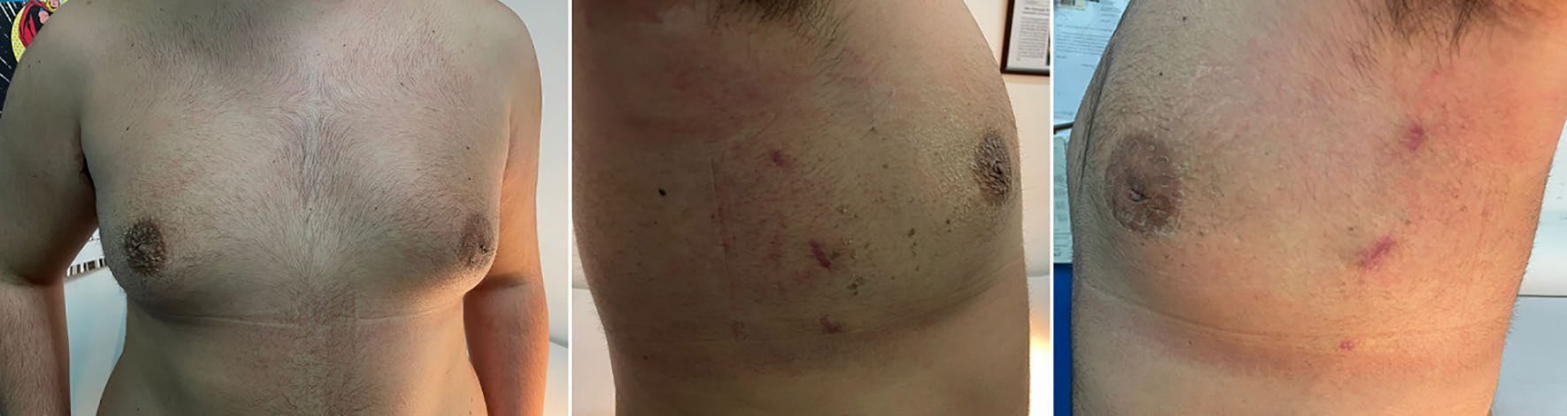
Aesthetic results at 2 months postoperatively

With respect to the aesthetic outcomes, they were very good in 21/24 (87.5%) patients. Minimal breast asymmetry between both sides was initially documented in 5 (20.8%) patients. It improved spontaneously in 2 (8.3%) boys after 2 years follow-up and persisted in 3 (12.5%), who did never perceive it as bad aesthetic outcome (Fig. 8). One nipple appeared laterally displaced and needed massages to free subcutaneous adhesions. No nipple retraction was observed. Furthermore, evaluating surgeons observed that the scars remained hyperchromic for about 2 months postoperatively and returned to the normal skin color following 8–9 weeks. The mean patients’ satisfaction score was 4.6 (range 3–5).

Operative results, complications, and aesthetic outcomes are reported in Table 2.

**Table 2:** Operative results, complications, aesthetic outcomes

Operative results	
Mean operative time unilateral PESMA, min (range)	87 (98–160)
Mean operative time bilateral PESMA, min (range)	160 (140–250)
Conversion to open, *n* (%)	1 (4.2)
Mean weight of resected fibrous glandular tissue, gr (range)	48.3 (10–97)
Mean aspirated volume of chest fat, mL (range)	240 (70–400)
Mean blood loss intra-operatively, mL (range)	15.5 (5–60)
Mean drainage volume post-surgery, mL (range)	68.5 (15–210)
Mean drainage time post-surgery, days (range)	1.8 (1–3)
Mean length of stay, days (range)	2.2 (1–4)
Complications	
Burns, *n* (%)	4 (16.7%)
Subcutaneous seroma, *n* (%)	1 (4.1%)
Hematoma, *n* (%)	0
Aesthetic outcomes	
Transient breast asymmetry, *n* (%)	2 (8.3%)
Persistent breast asymmetry, *n* (%)	3 (12.5%)
Nipple lateral displacement, *n* (%)	1 (4.1%)
Nipple retraction, *n* (%)	0
Mean patients’ satisfaction score, *n* (range)	4.6 (3–5)

## Discussion

Surgery is considered the treatment of choice in male adolescents who present persistent breast enlargement after a period of observation of at least 12 months, breast pain or tenderness, and/or significant psychosocial distress and unsatisfactory body image, sometimes leading to the avoidance of those activities in which the chest is exposed (i.e., swimming, gym) [20].

According to the current literature, three main surgical approaches are available: liposuction alone, open surgery via sub-nipple incision, and endoscopic surgery [11–13]. Liposuction is considered a simple procedure, requiring only liposuction device. However, it is a blinded operation, with potential risk of pectoralis major or, more rarely, intrathoracic injury. Moreover, liposuction allows the removal of adipose tissue, but the gland remains intact. Thus, liposuction alone should be indicated for selected cases of adipomastia.

On the contrary, open surgery is effective to remove the hyperplastic gland and/or redundant skin but the surgical incisions result in unpleasant peri-areolar scarring [12, 13].

With the improvement of technology, endoscopy has been introduced as a minimally invasive alternative to open surgery for surgical treatment of gynecomastia [21, 22]. Its main advantage is the combination of liposuction and vision-guided gland resection. After the description of a novel endoscopic technique for the evaluation of the axilla in breast cancer patients by Tsangaris et al. in 1999 [23], the endoscopic approach was studied and applied in many breast pathology surgeries [24–26], until the first report of 65 gynecomastia cases treated with endoscopic subcutaneous mastectomy was published by Fan et al. in 2009 [15].

Our preliminary experience of 12 patients treated with pediatric endoscopic subcutaneous mastectomy (PESMA) was published in 2019 [18]. Over the last years, increased laparoscopic practice allowed us to modify and further improve the surgical technique. We herein aimed to standardize the main steps and outline the pearls and tricks of this procedure.

Firstly, correct patient installation, in semi-seated position with the ipsilateral arm or both the arms abducted at 145 degrees and fixed on headframe, was crucial to have good exposure of the operative field throughout the procedure and to check the symmetry of both sides at the end, thus improving the aesthetic results. This was especially important in case of adipomastia or adipo-gynecomastia.

Secondly, creation of proper working space was paramount before dissection phase. Sufficient liposuction helped create an adequate operation space and simplified subsequent endoscopic subcutaneous mastectomy. The liposuction phase aimed at removing some subcutaneous and retromammary adipose tissue and defining the glandular tissue. The endpoint of the liposuction was decided by the pinch thickness of breast tissue when the fibrous gland could be clearly seen and felt. The surgeon should avoid performing liposuction too close to the skin and NAC to avoid ischemia or skin injury.

To improve creation of working space, we recently adopted a home-made glove balloon filled with saline and inserted within the cavity. This tool was very helpful to favor Cooper ligament collapse and create adequate operative space, reducing bleeding and shortening the operative time of about 30 min in bilateral procedures.

In our experience, surgical performance significantly improved using Harmonic Scalpel, that provided more precise tissue dissection and meticulous hemostasis, preventing hematoma formation postoperatively.

During the gland resection, a key point was to leave about 5–10 mm of gland thickness behind the NAC to prevent its retraction, but this parameter was difficult to assess with endoscopic approach. During this phase, the operating surgeon should keep nondominant hand on the nipple to control the thickness of the flap and feel the tip of the sealing device through the transilluminated skin. Before ending the procedure, the symmetry of the NAC on both sides must be carefully checked. In case of evident asymmetry, adjunctive gland resection or second liposuction may be performed to improve the operative results.

For adequate pre-operative planning, it is worthwhile to obtain MRI of the breasts to assess the quantity of fatty tissue, the size and morphology of the gland and to make a differential diagnosis between gynecomastia, adipomastia, and/or adipo-gynecomastia (Fig. 1). This differentiation could help decision-making process about the surgical strategy: liposuction alone in case of adipomastia, subtotal mastectomy in case of gynecomastia, or combination of both in case of adipo-gynecomastia. Moreover, as already reported in the literature [12], we believe that the combination of PESMA and liposuction is inevitable as almost all types of gynecomastia consist of both glandular and lipomatous hypertrophy.

There is a current trend toward reducing the number of surgical incisions. Yang et al. [17] performed endoscopic subcutaneous mastectomy through a 3-cm axillary single incision, which was used for the injection of lipolysis solution, the insertion of multiport liposuction cannula and endoscopic instruments, the removal of the fibrous tissue en bloc, and the implantation of drainage tubes.

Our technique adopted three surgical skin incisions (0.5 cm; 0.5 cm; 1.0–1.2 cm) on the mid-axillary line. Aesthetic results were very good in 87.5% of our patients. All boys were extremely satisfied of the final cosmetic appearance, even those presenting persistent minimal breast asymmetry. For this reason, particular attention must be paid to the informed consent. Before surgery, patients should be carefully informed about all possible post-operative complications, such as nipple malposition, breast asymmetry, burns, hypertrophic or cheloid scars, depigmentation, and the possibility of additional surgeries.

Main limitations of the present study are the small patient cohort and the relatively short follow-up period. Furthermore, no reliable assessment tool to evaluate the post-operative aesthetic results was available, so the observer assessment method was adopted to strengthen the validity and reliability of the results.

Based on this preliminary experience, PESMA combined with liposuction was feasible and safe for surgical treatment of gynecomastia in this selected cohort of patients. Although challenging, this procedure provided good aesthetic results, with no scars on the anterior thoracic wall. Standardization of the operative technique was a key point for successful outcome.

## Supplementary Information

Below is the link to the electronic supplementary material.

Supplementary file1 (MP4 257862 kb)
